# Beneficial and biocontrol effects of *Trichoderma atroviride*, a dominant species in white birch rhizosphere soil

**DOI:** 10.3389/fmicb.2023.1265435

**Published:** 2023-10-30

**Authors:** Kuo Liu, Yu-Zhou Zhang, Hua-Ying Du, Zhi-Ying Wang, Pei-Wen Gu, Zhi-Hua Liu, Ze-Yang Yu

**Affiliations:** ^1^School of Agriculture, Ningxia University, Yinchuan, Ningxia, China; ^2^Ningxia Forest Disease and Pest Control and Quarantine Station, Yinchuan, Ningxia, China; ^3^School of Forestry, Northeast Forestry University, Harbin, Heilongjiang, China; ^4^College of Forestry, Shenyang Agricultural University, Shenyang, Liaoning, China

**Keywords:** *Trichoderma* identification, resistance induction, pioneer tree species, growth promotion, soil improvement

## Abstract

White birch (*Betula platyphylla* Suk.) is a typical pioneer tree species that is important in forest restoration in northern China, Japan, and Korea. In the present study, 37 isolates were obtained from *B. platyphylla* rhizosphere soils in Heilongjiang Province; they were identified as *T. pleuroticola* (3 isolates), *T. virens* (2 isolates), *T. hamatum* (8 isolates), *T. atroviride* (21 isolates, dominant species) and *T. asperelloides* (3 isolates). Stress tolerance tests (salt, alkali, and nutritional stress that simulated saline alkali or barren soil) and confrontation assays (with four pathogens) were performed to determine which isolates had good biocontrol ability in barren soil; the results show that *T. atroviride* was outstanding. Then, in order to determine the effect of *T. atroviride* on plants and soil, *Gynura cusimbua* seeds were sown and treated with a *T. atroviride* spore suspension, as was unsown soil. The seedlings treated using *T. atroviride* had significantly greater height, stem diameter, soluble protein content, soluble sugar content, and malonaldehyde (MDA) content and their catalase (CAT) activity was also significantly increased. In addition, when the plants were inoculated with *Alternaria alternata*, the plants treated using *T. atroviride* had stronger CAT activity, significantly higher soluble protein content and soluble sugar content, and significantly lower MDA content, which indicates stronger resistance and less injury caused by the pathogen. In addition, *T. atroviride* not only increased the content of available nitrogen and available phosphorus in the soil, but also promoted *G. cusimbua* seedlings’ absorption of available nitrogen and available phosphorus. Thus, the characteristics of *T. atroviride* may make it the main factor that helps *B. platyphylla* colonise cut-over lands. *T. atroviride*, a promising biocontrol candidate, can be used in agriculture and forestry.

## 1. Introduction

*Trichoderma* spp. are important biocontrol agents worldwide because they can inhibit the growth of pathogens, promote plant growth, and induce plant resistance (Poveda, 2021). The pathogen inhibition mechanisms of *Trichoderma* were found to be competition, antibiotism, and mycoparasitism, while the plant benefit mechanisms of *Trichoderma* were found to be growth promotion and plant defence response induction (Poveda, 2021). Much research has reported the biocontrol functions of *Trichoderma*; one study found that *T. harzianum* and *T. viride* could suppress 15 strains of *Alternaria alternata* (Uniyal and Singh, 2017). *T. atroviride* isolated from wheat straw can effectively inhibit *Fusarium graminearum* and can produce xylanases linked to the colonisation of *F. graminearum* (Cabrera et al., 2020). In addition, potatoes treated with *T. asperellum* TaspHu1 grew better and had stronger resistance to *A. alternata* (Yu et al., 2021b). *T. virens* and *T. atroviride* promoted the formation of lateral roots in *Arabidopsis*, which resulted in improved nutrient uptake capacity and an increase in the biomass of the roots and shoots (López-Bucio et al., 2015). *T. atroviride* enhanced the resistance of *Populus*, as the leaves inoculated with pathogens showed smaller specks (Raghavendra and Newcombe, 2013).

However, the effect of biopesticides made from *Trichoderma* spp. is influenced by complicated environments, and efficiency loss caused by location, temperature, humidity, or nutrients is a common problem with biocontrol agents (Mukherjee et al., 2012). A good way to solve this problem is by continuously collecting and filtering robust *Trichoderma*, which may rely on natural mutation and natural selection. The collection of *Trichoderma* is performed globally, and 320 *Trichoderma* strains were isolated in fields and identified across 71 species (Braithwaite et al., 2017). Ten *Trichoderma* strains were isolated in *Juglans mandshurica* rhizosphere soil and identified as four species, out of which *T. asperellum* TaspHu1 showed outstanding biocontrol potential (Yu et al., 2021b).

*Betula platyphylla*, a typical pioneer tree species, is often the first to colonise cut-over lands and lands affected by forest fire or typhoon damage and forms its population below the layer of the coniferous belt in the forest vertical distribution. It is thought that this species plays an important role in the secondary succession process (Wu et al., 2002), which is also important in forest restoration. As one species of mycorrhizal fungi, *Trichoderma* may play an important role in protecting or promoting the growth of *B. platyphylla*, and when we need to plant white birch for forest restoration, the addition of some *Trichoderma* may significantly increase plant survival rates. Therefore, the investigation and collection of *Trichoderma* in *B. platyphylla* rhizosphere soil is necessary and useful for application in forest restoration or incult soil. In the present study, *Trichodema* strains were isolated from 13 *B. platyphylla* rhizosphere soil samples and identified. The biocontrol potential of these *Trichoderma* isolates was measured, including stress resistance, pathogen inhibition, growth promotion, and effect on soil. Based on these measurements, the *Trichoderma* distribution in *B. platyphylla* was roughly revealed, and the relevant strains for future use in forestry restoration were evaluated.

## 2. Materials and methods

### 2.1. Isolation of *Trichoderma*

*Trichoderma* fungi were isolated from 13 soil samples (each sample was approximately 100 g) of white birch rhizosphere soil (at a depth of 5–15 cm). Of these soil samples, 10 were collected from planted forest at the forest farm of Northeast Forestry University and three were collected from the natural forest of Maoer Mountain (126.63°E, 45.72°N). From these soil samples, five 5 g subsamples of each sample were dissolved in 100 mL of sterile water, shaken well using a muddler, and made into stock solution, which was diluted to four concentrations (1/10, 1/100, 1/1000, and 1/1000). Each concentration of the solution (200 μL) was plated onto potato dextrose agar medium (PDA medium) in a 9 cm Petri dish and cultured at 28°C for 24–96 h. During this period, the Petri dishes were observed every 24 h, and the *Trichoderma* colonies were transferred to a new PDA medium for purification culture (Zhou et al., 2019).

### 2.2. Morphological and molecular identification of *Trichoderma*

The purified *Trichoderma* were inoculated on a PDA plate medium and cultured in the dark for 7 days at 28°C to observe their macroscopic morphology; images of the colonies were obtained. Some PDA medium blocks (their length, width, and height were approximately 0.5 cm × 0.5 cm × 0.1 cm) were prepared using a sterile blade and placed on a glass slide under aseptic conditions. Then, the *Trichoderma* spores were inoculated on the PDA medium block and the aseptic coverslip was covered to make hyphae grow on the coverslip. The *Trichoderma* fungi were cultured in the dark at 28°C for 48–72 h, and the relative humidity was 60%. The hyphae were stained with a blue dye (biohao Biotechnology Co., Ltd., Beijing, China, Catalog No. C0710) to observe and record their morphology and that of their spores under a microscope (Leica DM750, Wetzlar, Germany). Morphological identification relied on the descriptions found in previous research (Yang, 2009, in Chinese).

One isolate in each species identified by morphology was randomly chosen for molecular identification. The purified *Trichoderma* was inoculated in potato dextrose (PD) liquid medium and shaken at 28°C at 180 rpm for 48 h. The mycelia were filtered and collected using eight layers of gauze for DNA extraction using a DNA extraction kit (OMEGA Biotek, Beijing, China). Then, the extracted DNA were used as a template to amplify the ITS (internal transcribed spacer) region and *tef1-α* (translation elongation factor-1α) region; the primers were designed with reference to previous studies (Dou et al., 2019), The PCR products were purified using a gel extraction kit (Promega, Madison, WI, USA, Kit No. A9281) and subjected to direct automated sequencing using fluorescent terminators using an ABI 377 Prism Sequencer (Sangon Biotech, Shanghai, China). The sequencing results were compared and identified using the website of the Center for Culture Collection of *Trichoderma* (CCTC), Shanghai Jiaotong University.^1^ Other recommended reference *Trichoderma* gene sequences were selected from the database (Dou et al., 2019), and a phylogenetic tree was constructed using the maximum likelihood (ML) method, with 1,000 bootstrap replications in the MEGA 7.0 (Kumar et al., 2016) package. After identification, the sequences were submitted to Genbank.^2^ The *Trichoderma* isolates that underwent molecular identification were subjected to further analyses.

### 2.3. Stress resistance and confrontation assay of *Trichoderma*

To investigate the tolerance of the *Trichoderma* isolates to a harsh environment (saline alkali soil or barren soil), a culture medium simulating a harsh environment was established. Salt stress, alkali stress, and nutrition stress experiments were created using a diluted PDA medium (diluted to 1/2, 1/4, 3/4, 1/8, 1/16, or zero, only made up of agar and water), a concentrated PDA medium (concentrated to 2-fold or 4-fold), a PDA medium to which was added NaCl (to generate final concentrations of 2, 4, 6, 8, and 10%, w/v, g/mL), or a PDA medium to which was added NaHCO_3_ (to generate final concentrations of 0.1, 0.4, 0.7, 1, and 1.3%, w/v, g/mL). Then, 3 μL of spore suspension (10^6^ spores/mL) was pipetted out and inoculated into the salt stress, alkali stress, and nutrition stress media, after which the Petri dishes were left to stand for 5–10 min and then sealed using parawax film. The *Trichoderma* were cultured at 28°C and the colony radii was measured using a ruler at 48 h.

In order to analyse the antagonism of the *Trichoderma* isolates, dual culture was performed between *Trichoderma* and phytopathogens on a PDA medium. Purified cultured *Trichoderma* and *Cytospora chrysosperma*, *Botrytis cinerea*, *A. alternata*, and *Fusarium oxysporum* were prepared as inocula. A 5 mm diameter mycelial disc of each *Trichoderma* isolate was added to the periphery of a new aseptic Petri dish; opposite this placement, a 5 mm diameter mycelial disc of the phytopathogen was positioned. Each dual culture was set up with three replicates. The plates were cultured for 12 days, and a modified resistance evaluation was performed based on a previously described protocol (Popiel et al., 2008), which used the following scoring system: +8 meant the antagonist had fully grown on the plate, completely covering the plate and the phytopathogen; +6 meant the antagonist occupied 85% of the plate’s surface area; +4 meant the antagonist occupied 70% of the plate’s surface area; and 0 meant the antagonist occupied 50% of the plate’s surface area.

### 2.4. Influence of *Trichoderma atroviride* on *Gynura cusimbua*

A spore suspension of *T. atroviride* was prepared to a concentration of 10^7^ spores/mL. Twenty seeds of *Gynura cusimbua* (bought from the Taobao online shopping platform^3^) were planted in each pot (size: L × W × H = 40 cm × 28 cm × 6 cm); for the treatment group, 800 mL of spore suspension was evenly poured into the soil before planting, while for the control group, 800 mL of water was evenly poured into the soil before planting. Three replicates were performed. The seedlings were cultivated under a 16 h light/8 h dark photoperiod (under a daylight lamp) and 40% relative humidity and watered every 10 days with 500 mL of additional water. The germination rates and germination times of the plants were recorded. At 30 days after seed sowing, the stem height and stem diameter were recorded, and the leaves of three randomly chosen seedlings were harvested and stored at −80°C for malondialdehyde (MDA), catalase (CAT) activity, soluble sugar content, and soluble protein content analyses (Jiancheng Bioengineering Institute, Nanjing, Jiangsu, China, Kit A003-1-2, Kit A00-1-1, Kit A145-1-1 and Kit A045-2-2).

Then, the 6–10 randomly chosen leaves of other plants in the control group and treatment group were pierced (4 times on each leaf) and inoculated with *A. alternata*, marked as C + A (control + *A. alternata*) and T + A (treatment + *A. alternata*). After 12 days, the morbidity was observed, and the MDA, CAT activity, soluble sugar content, and soluble protein content were also analysed.

Approximately 200 g of soil samples in the control group and treatment group were collected, marked as the P group (the soil in which *G. cusimbua* was planted) and T + P group (the soil watered with the spore suspension of *T. atroviride* in which *G. cusimbua* was planted), and stored at −20°C for further analysis.

### 2.5. Influence of *Trichoderma atroviride* on soil nutrition

Six pots (size: L × W × H = 40 cm × 28 cm × 6 cm) containing the same soil as that in the experiment above were prepared; for three of these, 800 mL of spore suspension of *T. atroviride* was evenly poured into the soil, while for the other three, 800 mL of water was evenly poured. The pots were placed under a 16 h light/8 h dark photoperiod (under a daylight lamp) and 40% relative humidity and watered every 10 d with 500 mL of additional water for 30 days (three times). Then, the soil samples (approximately 200 g) were collected, marked as the T group (the soil watered with the spore suspension of *T. atroviride*) and the CK group (the soil watered with water) and stored at −20°C for further analysis. The available nitrogen and available phosphorus of the soil in the P, T + P, T, and CK groups were analysed according to previous research (Bardgett et al., 2007; Zhou et al., 2020).

### 2.6. Statistical analysis

All experimental data were subjected to an analysis of variance (ANOVA) using SPSS software v19.0 (IBM Corp., Armonk, NY, USA). The statistical significance of the differences between the means was determined using Duncan’s multiple-range tests and an independent-sample T test (*p* < 0.05).

## 3. Results

### 3.1. Morphological and molecular identification of *Trichoderma*

**Figure 1:**
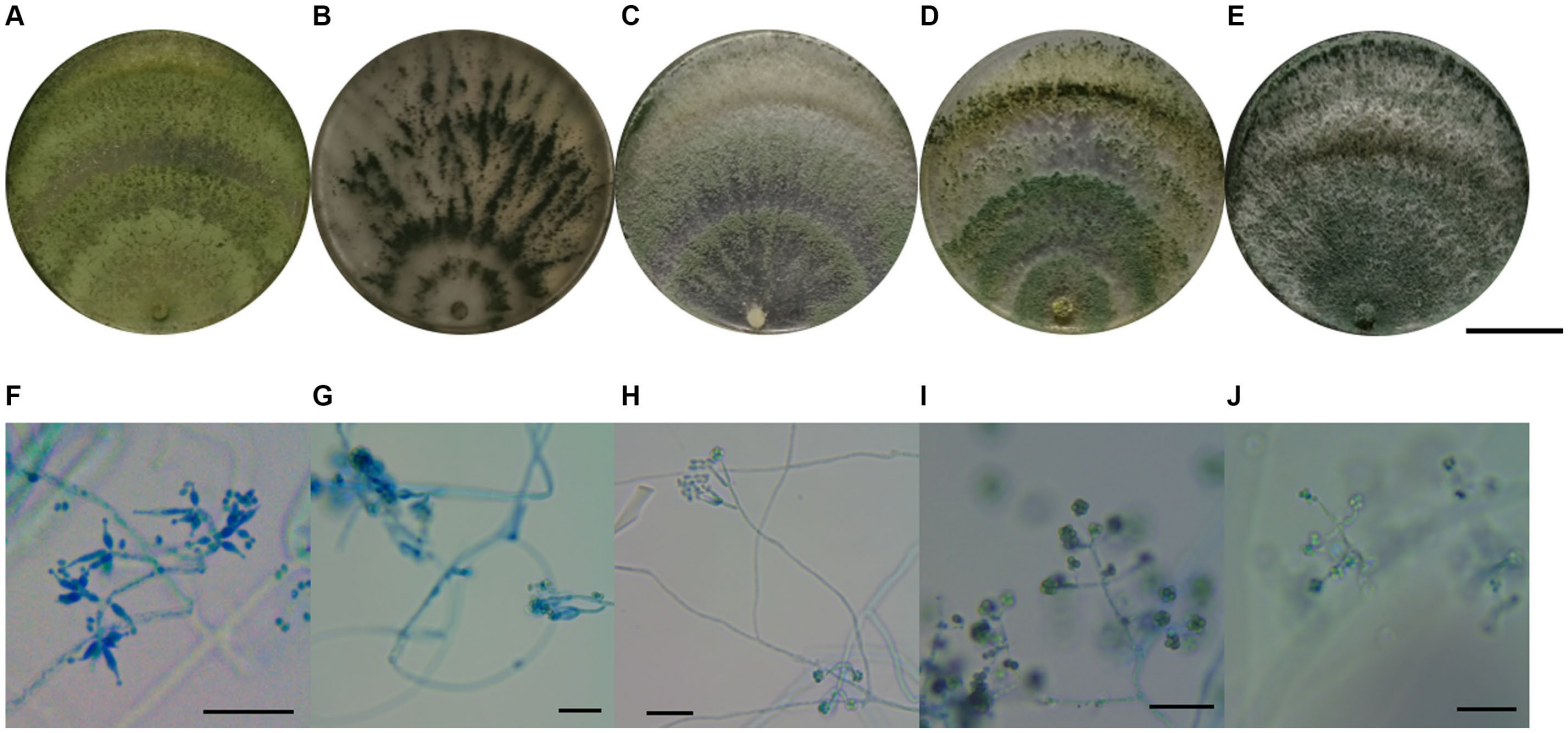
Morphology of *Trichoderma*. **(A)** Macro-morphology of *T. pleuroticola*. **(B)** Macro-morphology of *T. virens*. **(C)** Macro-morphology of *T. hamatum*. **(D)** Macro-morphology of *T. atroviride*. **(E)** Macro-morphology of *T. asperelloides*. **(F)** Micro-morphology of *T. pleuroticola*. **(G)** Micro-morphology of *T. virens*. **(H)** Micro-morphology of *T. hamatum*. **(I)** Micro-morphology of *T. atroviride*. **(J)** Micro-morphology of *T. asperelloides*. Ruler in **(A–E)** = 3 cm, ruler in **(F–J)** = 50 μm.

**Figure 2:**
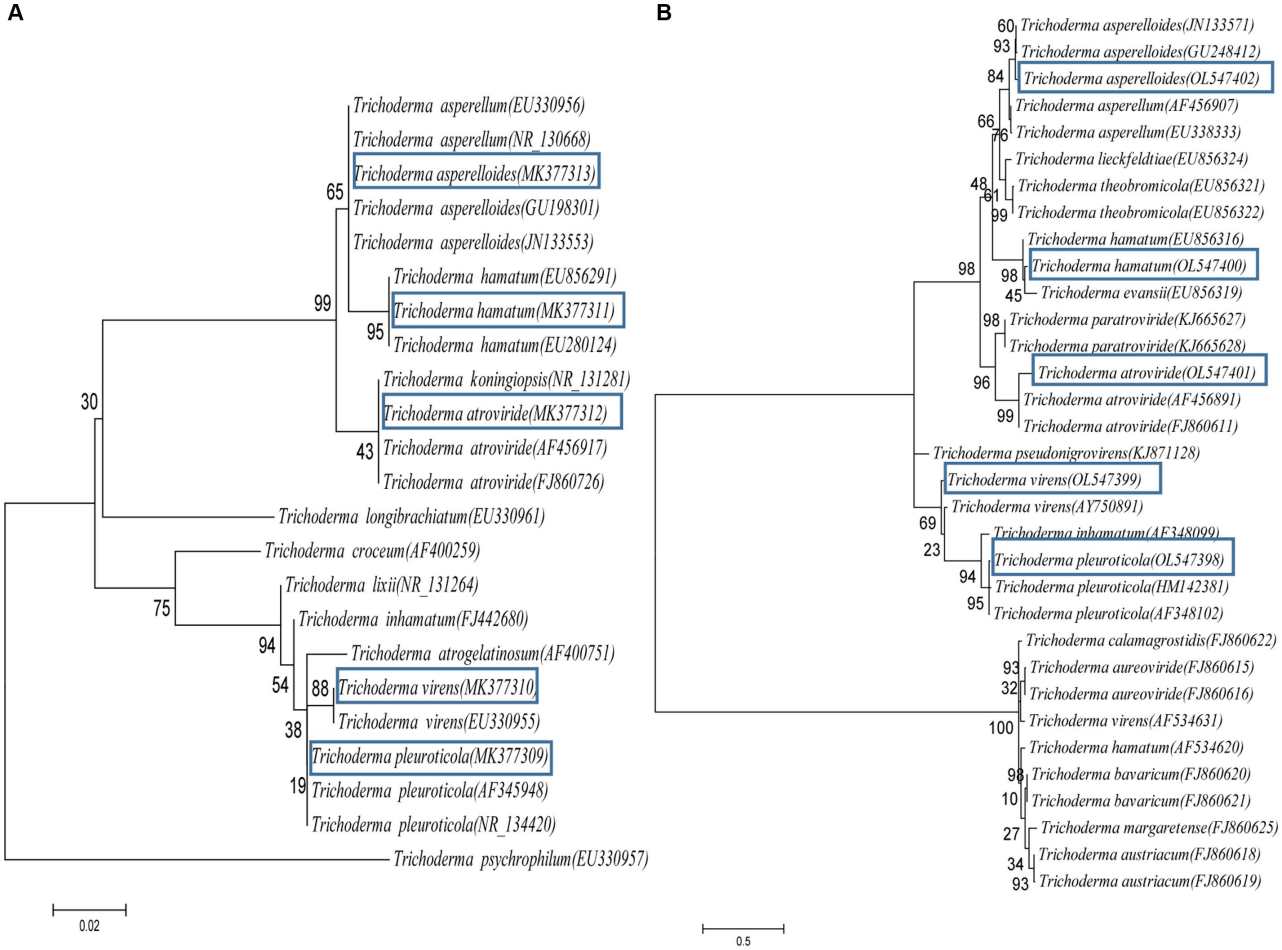
Phylogenetic analysis based on ITS and *tef1-α* sequences of *Trichoderma* strains. **(A)** Phylogenetic tree constructed using ITS sequences. **(B)** Phylogenetic tree constructed using *tef1-α* sequences. The accession numbers of the sequences are provided in brackets.

**Figure 3:**
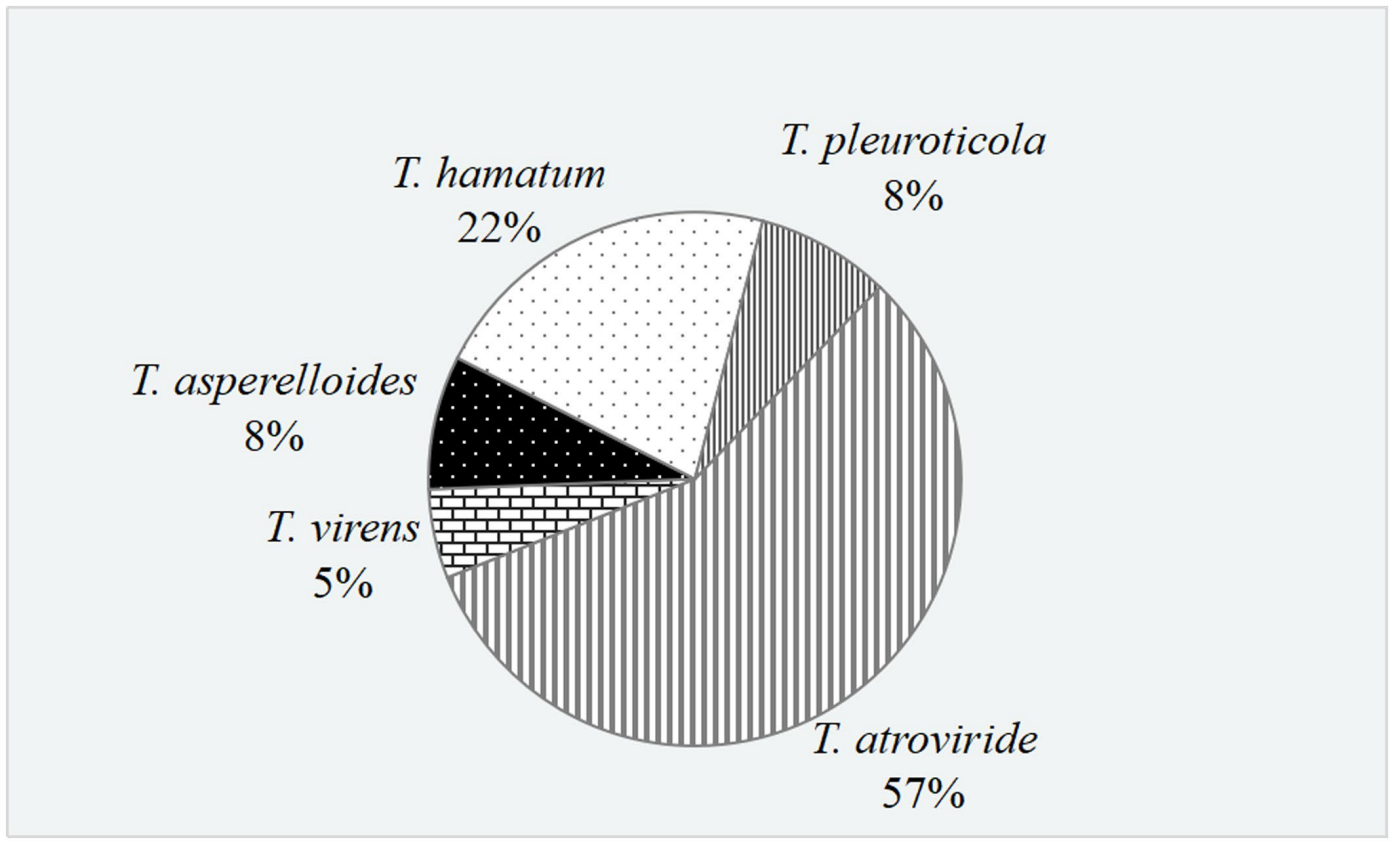
Proportion of *Trichoderma* in the rhizosphere soil of white birch.

In total, 37 *Trichoderma* isolates were obtained from 13 soil samples, and 5 species were identified based on morphological comparison (Figure 1): *T. pleuroticola* (3 isolates), *T. virens* (2 isolates), *T. hamatum* (8 isolates), *T. atroviride* (21 isolates), and *T. asperellum* (3 isolates). One isolate was randomly selected from each *Trichoderma* species for further molecular identification. The ITS regions and *tef1-α* regions of these five *Trichoderma* isolates were amplified and sequenced. The sequences were by consulting the Center for Culture Collection of *Trichoderma* (CCTC), Shanghai Jiaotong University (see footnote 1). The alignment results show that most morphological identification was accurate, except for the *T. asperellum* sample, which was identified as *T. asperelloides* based on molecular characteristics. Phylogenetic trees were also constructed using the reference sequences recommended by the CCTC database (Figure 2); the results accompany the alignment results. Based on the above results, five species of *Trichoderma* were obtained: *T. pleuroticola*, *T. virens*, *T. hamatum*, *T. atroviride*, and *T. asperelloides*. The sequenced ITS and *tef1-α* sequences of the five *Trichoderma* isolates were submitted to Genbank (see footnote 2), and the accession numbers of these ITS sequences are MK377309 (*T. pleuroticola*), MK377310 (*T. virens*), MK377311 (*T. hamatum*), MK377312 (*T. atroviride*), and MK377313 (*T. asperelloides*). The accession numbers of the *tef1-α* sequences are OL547398 (*T. pleuroticola*), OL547399 (*T. virens*), OL547400 (*T. hamatum*), OL547401 (*T. atroviride*), and OL547402 (*T. asperelloides*). Among these isolated *Trichoderma*, the number of isolates of *T. atroviride* was the highest (21 isolates), accounting for 57% of the total isolated *Trichoderma*. *T. hamatum* followed with 8 isolates, accounting for 22% of the total. *T. asperelloides* and *T. pleuroticola* both had three isolates, accounting for 8% of the total. There were the least *T. virens* isolates, with two strains, accounting for 5% of the total (Figure 3).

### 3.2. Stress tolerance and pathogen inhibition of *Trichoderma* isolates

**Figure 4:**
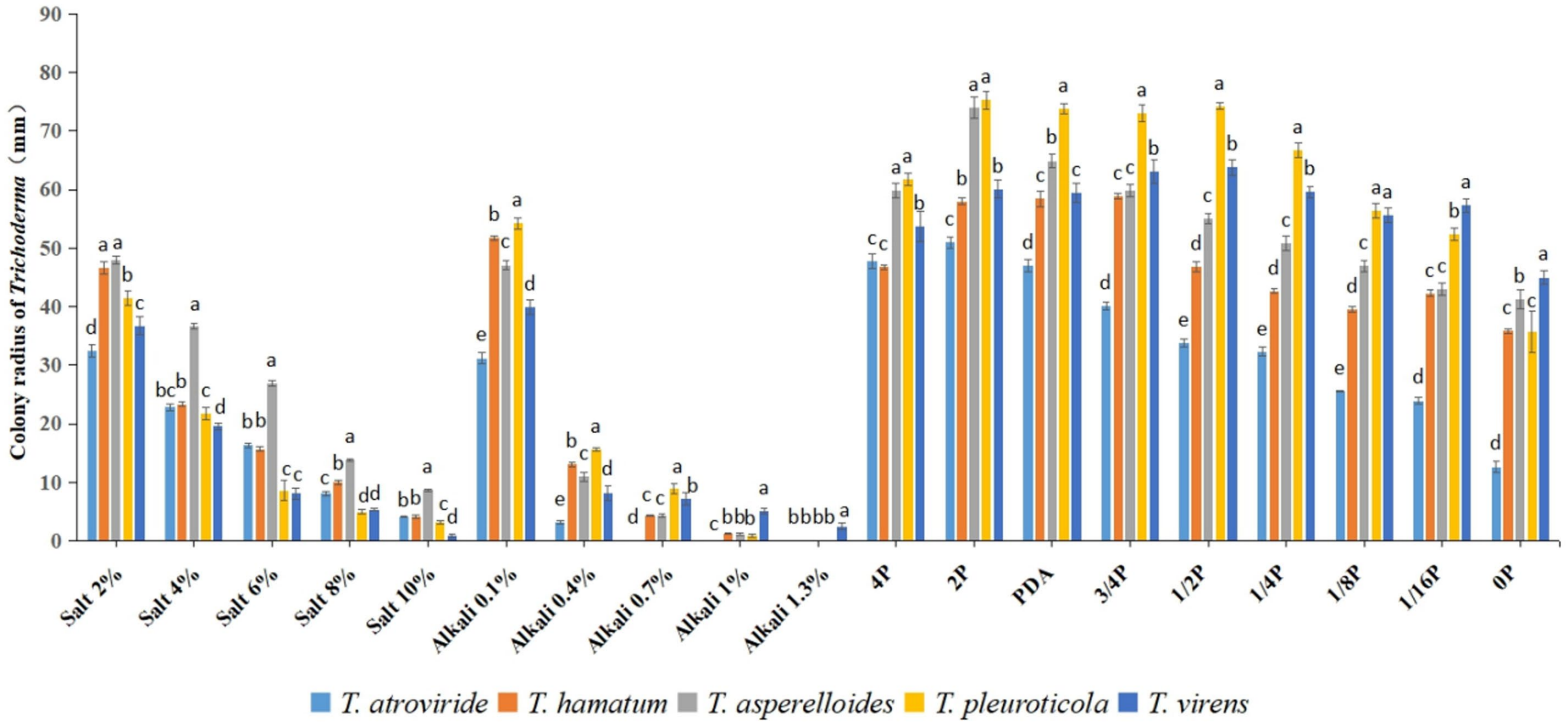
Radius of *Trichoderma* colonies in different conditions. All values represent the mean of three replicates ± standard error; in each condition, different letters denote a statistically significant difference according to a one-way ANOVA test (*p* < 0.05).

In this study, all five *Trichoderma* candidate strains for which molecular identification was performed could grow in the five concentrations of the salt stress medium, among which *T. asperelloides* grew better and had stronger salt stress adaptability, followed by *T. hamatum*, which also had relatively good salt stress tolerance. In the alkaline stress medium, *T. atroviride* could not grow when the alkali concentration reached 0.7% (weight/volume, g/mL), while *T. virens* grew slowly when the alkali concentration reached 1.3% (weight/volume, g/mL). *T. virens* and *T. pleuroticola* have better adaptability to alkali stress. In the media with different nutrient concentrations, *T. pleuroticola*, *T. virens*, and *T. asperelloides* grew better (Figure 4).

**Figure 5:**
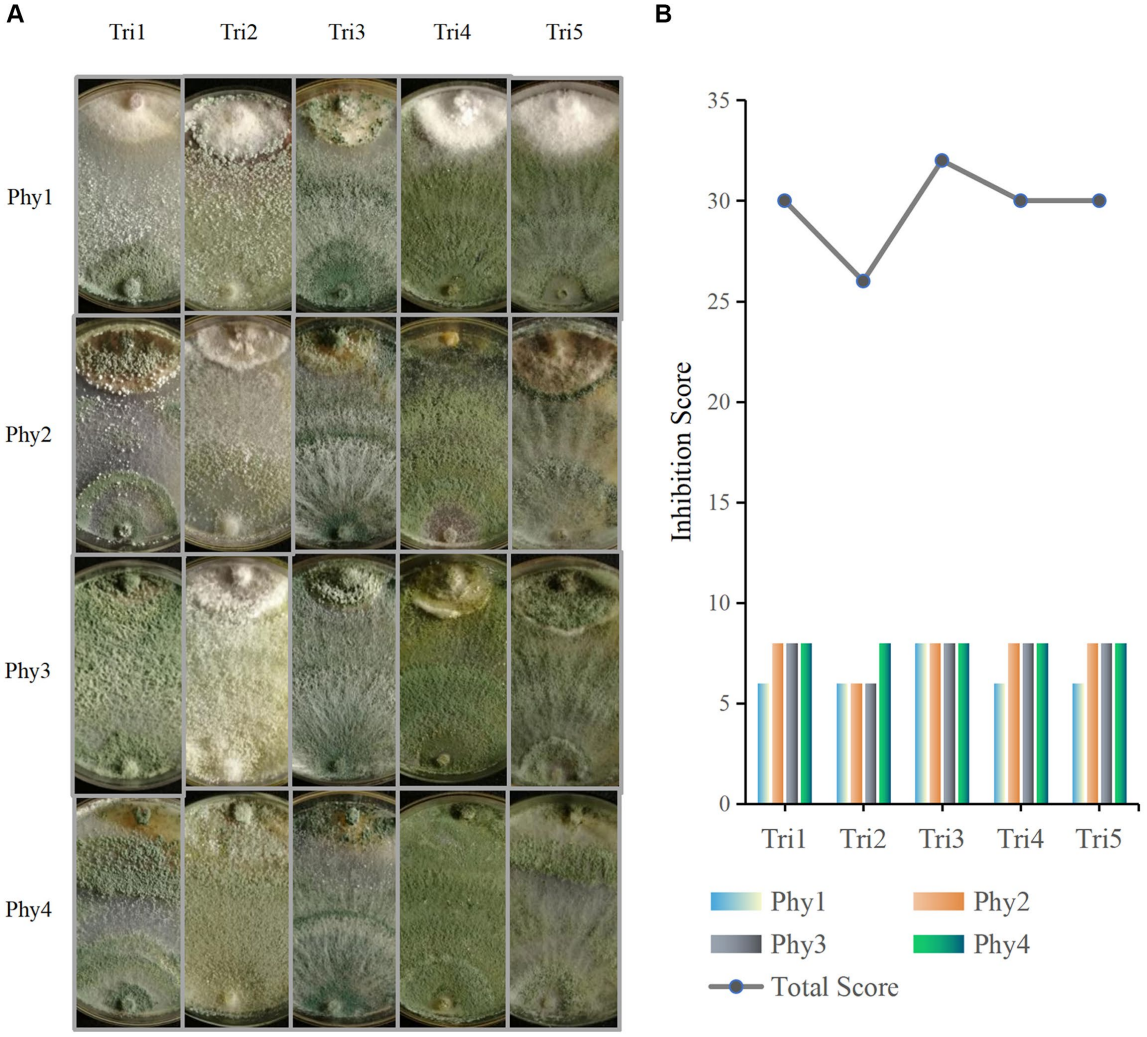
Phytopathogen inhibition ability of *Trichoderma* isolates. **(A)** Confrontation assay between *Trichoderma* (Tri) and phytopathogens (Phy); in the picture, *Trichoderma* are at the bottom and phytopathogens are at the top. **(B)** Pathogen inhibition ability evaluation. Tri1: *T. atroviride*; Tri2: *T. hamatum*; Tri3: *T. asperelloides*; Tri4: *T. pleuroticola*; Tri5: *T. virens*; Phy1: *F. oxysporum*; Phy2: *A. alternata*; Phy3: *B. cinerea*; Phy4: *C. chrysosperma*. Each dual culture was set up with three replicates.

The confrontation assay shows that the five *Trichoderma* isolates spread in the Petri dish rapidly reacted to and had varying degrees of antagonistic responses against *F. oxysporum*, *A. alternata*, *B. cinerea*, and *C. chrysosperma* (Figure 5). Among them, *T. asperelloides* could not only inhibit the growth of the four pathogens, but also parasitised the tested pathogens, occupying the entire plate; thus, it had the highest evaluation score (Figure 5B). With the exception of *F. oxysporum*, the other three pathogens were also parasitised by *T. atroviride*, *T. pleuroticola*, and *T. virens* (Figure 5A); thus, those species also have relatively high evaluation scores. *T. hamatum* could only parasitise *C. chrysosperma* but could not parasitise the other three pathogens (Figure 5A).

### 3.3. Influences of *Trichoderma atroviride* on *Gynura cusimbua*

**Figure 6:**
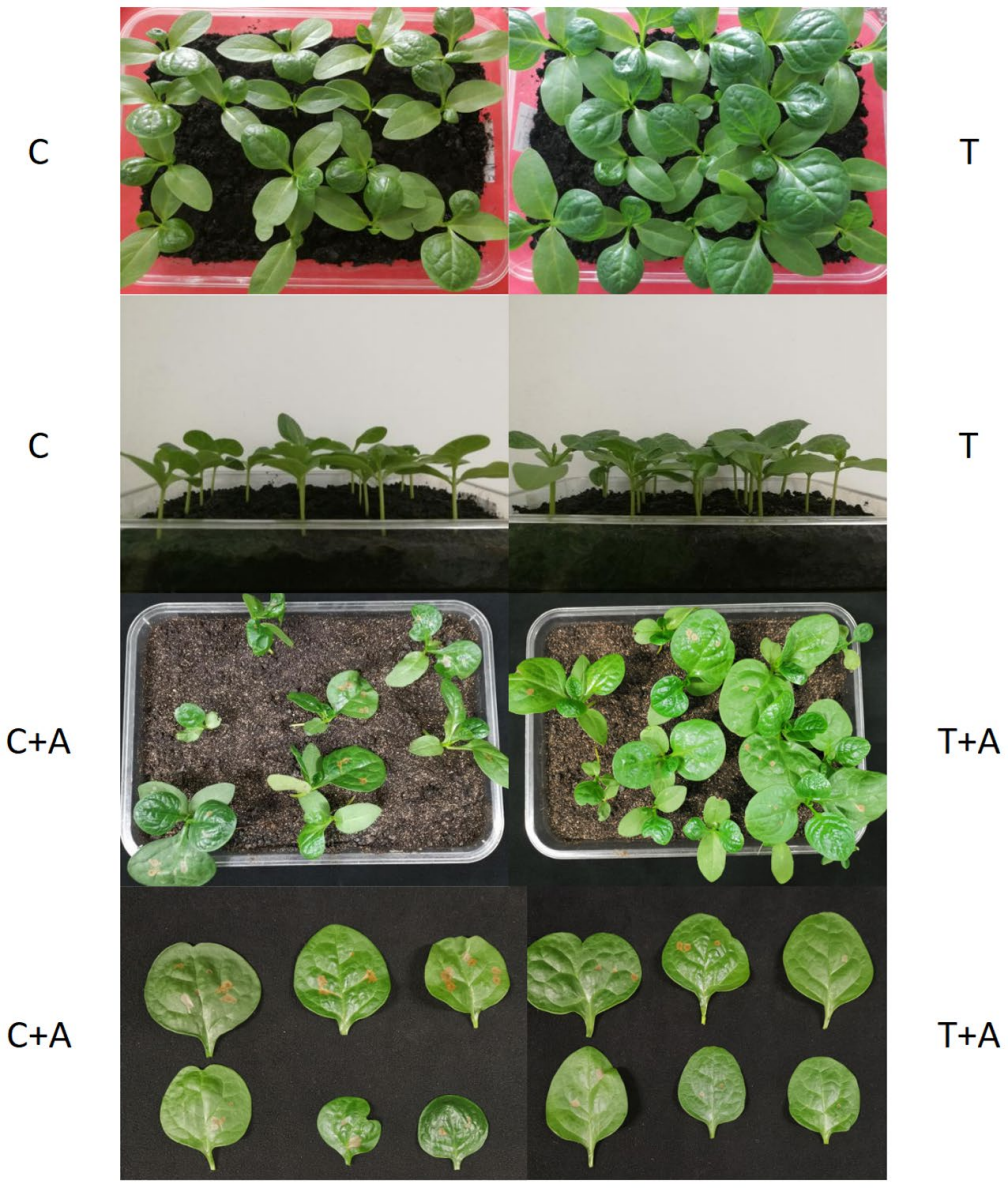
Growth of *G. cusimbua* at 30 days (left: control; right: treatment) and test of pathogen resistance ability. T represents *G. cusimbua* watered with a spore suspension of *T. atroviride*; C represents *G. cusimbua* watered with water; T + A represents *G. cusimbua* watered with a spore suspension of *T. atroviride*, then inoculated with *Alternaria alternata*; and C + A represents *G. cusimbua* watered with water, then inoculated with *A. alternata*.

In the treatment group (T), the seed germination rate of the treatment group was 90% and the average germination time was 102.3 h, while in the control group, the germination rate was 80% and the average germination time was 105.2 h; this difference was not significant (Table 1). Thirty days later, we found that the seedlings in the treatment group had better growth than those in the control group (Figure 6). The average plant height of the plants in the treatment group was 4.60 cm, significantly higher than that (4.12 cm) of the control group (*p* < 0.05). Similarly, the average stem diameter of the plants in the treatment group (5.98 mm) was also significantly higher than that (5.04 mm) of the control group (*p* < 0.05, Table 1). After inoculation with *A. alternata*, the height and stem diameter of plants did not significantly change in the treatment group (T + A) and control group (C + A).

**Table 1:** Physiological index of *Gynura cusimbua.*

	Malonaldehyde (μmol/100 g)	Soluble protein (μg/g)	Soluble sugar (mg/g)	Catalase activity (nmol/min/g)	Height (cm)	Stem diameter (mm)	Germination rate (%)	Mean germination time (h)
T	31.709 ± 0.542^c^	1555.458 ± 27.984^b^	1.5362 ± 0.050^b^	1053.63 ± 45.147^b^	4.60 ± 0.235^ab^	5.98 ± 0.20^a^	90	102.3 ± 19.8
Control	16.13 ± 0.418^d^	1161.634 ± 31.607^d^	1.3238 ± 0.045^c^	256.95 ± 37.86^d^	4.120 ± 0.149^b^	5.04 ± 0.15^b^	80	105.3 ± 21.9
T + A	35.797 ± 1.469^b^	1694.710 ± 15.204^a^	1.7677 ± 0.040^a^	1773.78 ± 97.064^a^	4.72 ± 0.217^a^	6.04 ± 0.16^a^		
C + A	39.077 ± 0.446^a^	1397.893 ± 12.891^c^	1.664 ± 0.037^a^	735.70 ± 40.554^c^	4.22 ± 0.140^b^	5.12 ± 0.18^b^		

All values represent the mean of three replicates ± standard error. T represents *G. cusimbua* watered with a spore suspension of *T. atroviride*; Control represents *G. cusimbua* watered with water; T + A represents *G. cusimbua* watered with a spore suspension of *T. atroviride*, then inoculated with *Alternaria alternata*; and Control + A represents *G. cusimbua* watered with water, then inoculated with *A. alternata*. Different letters denote statistically significant differences according to a one-way ANOVA test (*p* < 0.05).

Further, some physiological indices were measured. For the treatment group, the MDA was 31.709 μmol/100 g, significantly higher than the MDA of the control group (16.13 μmol/100 g, *p* < 0.05). The soluble protein for the treatment group was 1,555.458 μg/g, significantly higher than that of the control group (1,161.634 μg/g); the soluble sugar of the treatment group was 1.5362 mg/g, significantly higher than that of the control group (1.3238 mg/g, *p* < 0.05). The catalase activity showed similar results, with the activity in the treatment group (1,053.63 nmol/min g) significantly higher than that in the control group (256.95 nmol/min g, *p* < 0.05).

After inoculation with *A. alternata*, the MDA values in both T + A and C + A were increased, but the MDA in C + A increased more dramatically (from 16.13 μmol/100 g to 39.077 μmol/100 g) and was significantly higher than the MDA of the plants in T + A (35.797 μmol/100 g, *p* < 0.05). The soluble protein of the plants increased in both groups, its value in T + A (1,694.710 μg/g) being still significantly higher than that in C + A (1,397.893 μg/g, *p* < 0.05). The soluble sugar of the plants also increased in both groups, and its value in T + A (1.7677 mg/g) was also still significantly higher than that in C + A (1.664 mg/g, *p* < 0.05). The CAT activity in plants of both groups was increased, and its value in T + A (1,773.78 nmol/min g) was significantly higher than that in C + A (735.70 nmol/min g, p < 0.05). Meanwhile, the plants in the T + A group grew better and had less or smaller disease spots compared to the plants in C + A group, which indicates that *T. atroviride* improves the disease resistance of *G. cusimbua*.

### 3.4. Beneficial effects of *Trichoderma atroviride* on soil nutrients

**Figure 7:**
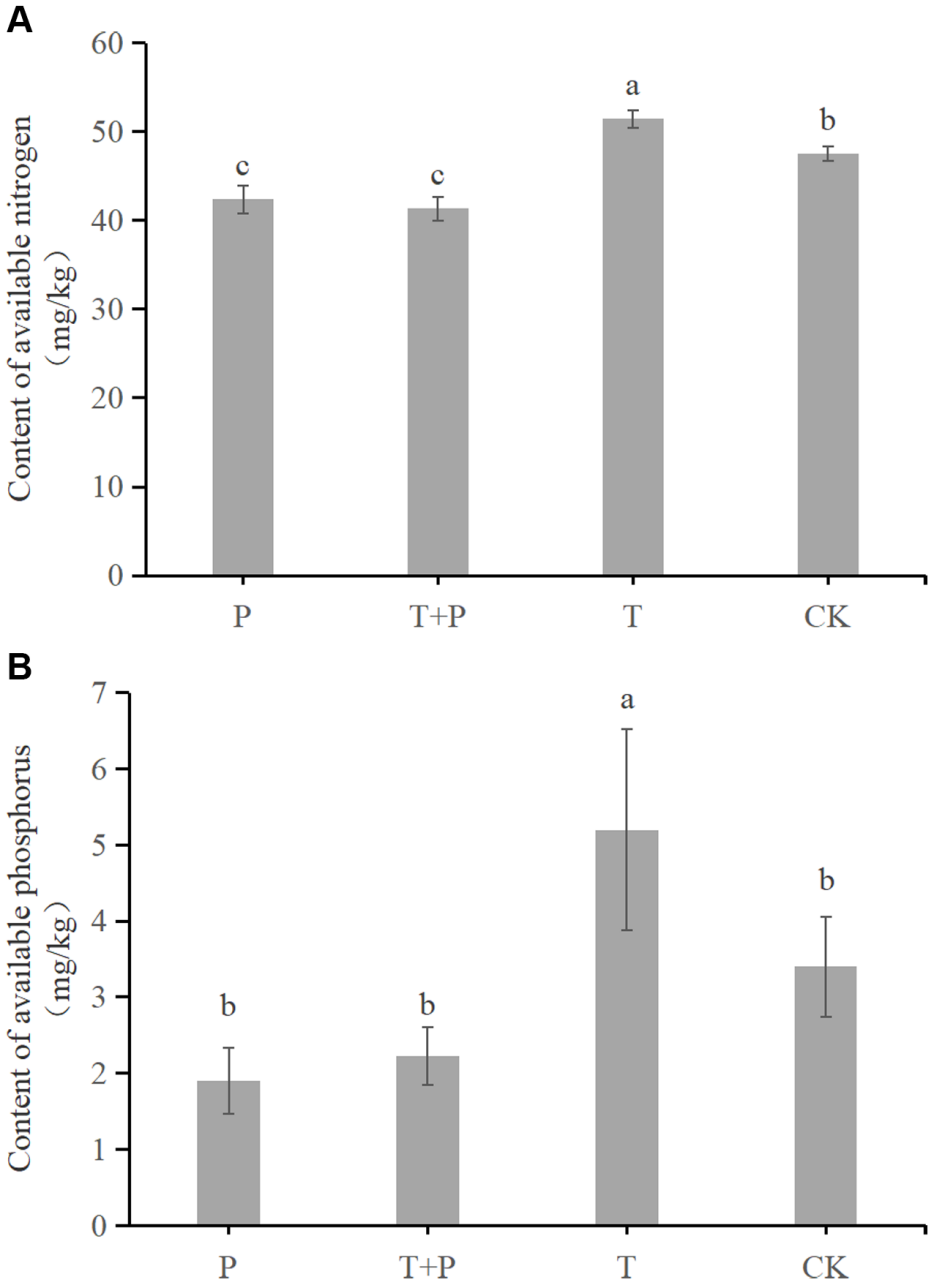
The influence on soil nutrients of *T. atroviride* and *G. cusimbua*. *(A)* Content of available nitrogen; **(B)** Content of available phosphorus. P: the soil in which *G. cusimbua* was planted; T + P: the soil watered with spore suspension of *T. atroviride*, in which *G. cusimbua* was planted; T: the soil watered with spore suspension of *T. atroviride*; CK: the soil watered with water. All values are the mean of three replicates ± standard error. Different letters denote statistically significant differences according to a one-way ANOVA test (*p* < 0.05).

The contents of available nitrogen and available phosphorus in the four groups were measured (Figure 7). The available nitrogen in the P group, T + P group, T group, and CK group was 38.7 mg/kg, 41.33 mg/kg, 51.43 mg/kg, and 47.5 mg/kg, respectively (Figure 7A). The available phosphorus in the P group, T + P group, T group, and CK group was 1.9 mg/kg, 2.33 mg/kg, 5.2 mg/kg and 3.4 mg/kg, respectively (Figure 7B). The results show that the use of *T. atroviride* significantly improves the content of available nitrogen and available phosphorus in soil. Meanwhile, compared with the soil in which *G. cusimbua* was not planted, the soil in which *G. cusimbua* was planted had significantly lower available nitrogen content (*p* < 0.05, Figure 7A) and lower available phosphorus (Figure 7B). Furthermore, the decrease in available nitrogen from T to T + P (10.1 mg/kg) was more than the decrease from CK to P (8.8 mg/kg); the decrease in available phosphorus from T to T + P (2.87 mg/kg) was more than the decrease from CK to P (1.5 mg/kg), which indirectly shows that *T. atroviride* promotes the absorption of available nitrogen and available phosphorus in *G. cusimbua*.

## 4. Discussion

*Trichoderma* spp. have been widely used in agriculture and forestry as biocontrol agents due to their good biocontrol ability (Gafur, 2023; Gafur et al., 2023). The collection and identification of *Trichoderma* resources has also become an important way to continuously explore high-quality *Trichoderma* in terms of biocontrol (Błaszczyk et al., 2011; Yu et al., 2021a,b). Dou et al. isolated 3,999 *Trichoderma* strains from four ecosystems (forest, grassland, wetland, and agro-ecosystem) in 28 provinces of China, and they were identified as 50 species. In Heilongjiang Province, they isolated 472 *Trichoderma* isolates and identified 23 species, of which *T. harzianum* was the main species, followed by *T. hamatum* and *T. atroviride* (Dou et al., 2019). In this study, among the *Trichoderma* isolated from the rhizosphere soil of *B. platyphylla* in Harbin, *T. atroviride* was the main species, accounting for 57% of the total number, followed by *T. hamatum*; *T. harzianum* was not isolated (Figure 3). The results are similar to those in previous research, where *T. atroviride* was the dominant species isolated from the rhizosphere soil of *J. mandshurica*, *Acer saccharum*, *Populus simonii*, and *Ulmus pumila* forests in Heilongjiang Province (Zhou et al., 2019). The similarity or difference in *Trichoderma* distribution may be caused by the differences in the rhizosphere environment, nutrition, and secondary metabolites of different plant species.

The identification methods in this study were combined morphological identification and molecular identification (Figures 1 2). Most of the *Trichoderma* isolated in this study were correctly identified via careful morphological identification and consistent with the molecular identification results, except one: *T. asperelloides* was wrongly identified as *T. asperellum*. The main reason for this is that the book we referenced was published in 2009 (Yang, 2009, in Chinese), but *T. asperelloides* was reported in 2010 (Samuels et al., 2010); in addition, Samuels (Samuels et al., 2010) reported that *T. asperellum* is morphologically indistinguishable from *T. asperelloides*. This indicates that morphological identification is insufficient for *Trichoderma* identification. In our opinion, morphological identification is an important skill for taxonomists; we do not suggest that researchers should abandon this skill, nor do we advise that molecular identification is the only way to identify *Trichoderma*. Furthermore, even though the cost of molecular identification has decreased, it is still costly to sequence each isolate for some researchers who are short of funds, which may limit the development of taxonomy. In addition, taxonomy is not an exact science: it is the classification of species based on characteristics, similarities, evolutionary history, and even particular lifestyles (Garnett et al., 2020; Lücking et al., 2020); therefore, we should carefully evaluate the decision to base classification solely on tiny differences in gene loci.

In order to filter potential biocontrol agents, resilience experiments and confrontation assays were performed (Figures 4 5). After comprehensively evaluating our five *Trichoderma* isolates for resilience and pathogen-inhibiting ability, *T. asperelloides* was outstanding above the other four *Trichoderma* isolates; this result is consistent with Ramírez-Cariño’s research that reported that *T. asperelloides* was able to exert an outstanding mycoparasitic effect on *A. alternata* and *F. oxysporum* in a dual confrontation assay by hyphal strangulation and penetration (Ramírez-Cario et al., 2020). Similar results have also been found for its sibling species, *T. asperellum*; TaspHu1 showed better biocontrol ability compared to other isolates (Yu et al., 2021b). However, inconsistent results in the laboratory and field have also been reported (Mukherjee et al., 2012; López-Quintero et al., 2013). Additionally, *T. atroviride* also has relatively good adaption to different conditions and good inhibition ability; *T. atroviride* grew fast and effectively inhibited the growth of the four tested phytopathogens and could also mycoparasitise three of the four phytopathogens, except for *F. oxysporum*. This result is similar to that of a previous study, in which *T. atroviride* isolated from organic composts was reported to successfully control the disease of *Clarireedia* spp., *Rhizoctonia solani*, and *Sclerotium rolfsii*, which showed 53.5, 69.3, and 43.5% less affected area, respectively, on *Agrotis stolonifera* compared to the control (Coelho et al., 2021). The number of *T. atroviride* isolates was the greatest and accounted for 57% of the total number, while the number of isolated *T. asperelloides* accounted for only 8%. Therefore, considering that *T. atroviride* is widely distributed in rhizosphere soils of *B. platyphylla*, it may have good ability in the field; thus, *T. atroviride* was further analysed. As an additional consideration, trees grow slowly and we did not have the ability to plant white birch, so we decided to plant vegetables to verify *Trichoderma*’s plant-promoting abilities.

That *Trichoderma* can promote plant growth has been widely reported; for example, in tomatoes treated using *T. atroviride* MUCL45632, the shoot height significantly increased from 46.8 cm to 48.5 cm and the root dry weight significantly increased from 1.3 g to 2.7 g (Colla et al., 2015). In our study, *T. atroviride* significantly increased the stem diameter from 5.04 mm to 5.98 mm and the shoot height also increased from 4.12 cm to 4.60 cm. MDA reflects the degree of cell damage. Ji et al. found that *Trichoderma* induced the expression of the MsERF105 gene of *Malus sieversii,* which led to a significant decrease in MDA after 72 h (Ji et al., 2021). Rawat et al. also found lower MDA accumulation in *Trichoderma*-treated plants, which might be the reason that the plants endured salt stress (Rawat et al., 2016). In our study, a similar result also appeared; *T. atroviride* reduced the content of MDA when *G. cusimbua* was inoculated with *A. alternata*. However, treatment using *T. atroviride* alone still significantly increased the content of MDA, which indicates that *T. atroviride* also slightly injures plants, which may trigger their defence response.

Soluble protein and soluble sugar play important roles in the response to stress as osmotic balancers (Hoekstra et al., 2001; Afzal et al., 2021). In poplars treated using *T. asperellum* T-Pa2, the contents of soluble protein and soluble sugar were significantly increased (Yu et al., 2021a). The contents of soluble protein and proline in tomatoes increased significantly, by 32.08 and 26.7%, respectively, after induction with *T. harzianum*, which enhanced the stress tolerance of the tomatoes. In our study, after treatment using *T. atroviride*, the content of soluble protein and soluble sugar in *G. cusimbua* were also significantly increased; meanwhile, after inoculating with *A. alternata*, *G. cusimbua* treated using *T. atroviride* expressed higher contents of soluble sugar and soluble protein compared to the control. Reactive oxygen species (ROS) increase when plants are under stress, and ROS damage the cell and its DNA (Zhao et al., 2019). Enzymes such as catalase (CAT) and superoxide dismutase (SOD) can “clear out” ROS; therefore, their enzyme activity reflects the ability to counter and eliminate ROS and also reflects the ability to adapt to stress (Zhao et al., 2019). *T. afroharzianum* T52 induced a 7.4-fold increase in CAT activity in *Syringa oblata*, while the H_2_O_2_ content decreased significantly to 0.82 μmol/g relative to 4.03 μmol/g in the control (Liu et al., 2020). In addition, poplars treated using *T. asperellum* T-Pa2 led to CAT significantly increasing to 3618.2 nmol/min/g compared to 2,926.62 nmol/min/g in the control (Yu et al., 2021a). In the present study, the plants treated with *T. atroviride* alone (T) or *A. alternata* alone (C + A) both improved in terms of CAT activity; however, the CAT activity of plants in T (1,053.63 nmol/min/g) was significantly higher than that of plants in C + A (735.70 nmol/min/g). Further, the plants treated using *T. atroviride* were more sensitive to *A. alternata* and had stronger responses to *A. alternata*, and the CAT activity in T + A (1,773.78 nmol/min/g) was significantly higher than in T or C + A. Therefore, we speculate that one of the reasons for the successful infection of pathogens is the failure to provoke sufficiently fast and strong defence responses in plants.

Strain *T. atroviride* MUCL45632 increased the nutrient levels of the plants, which caused increases in root and shoot weights (Colla et al., 2015). *T. asperellum* TaspHu1 promoted the absorption of available nitrogen in tomatoes, which caused increases in tomato growth; however, the strain did not influence the soil nitrogen content (Yu et al., 2021b). In the present study, *T. atroviride* significantly increased the content of available nitrogen and available phosphorus in the soil and also promoted the absorption of nutrients by *G. cusimbua*.

In conclusion, *T. atroviride* was found to be the leading species in *B. platyphylla* rhizosphere soil. It has a good effect on pathogen inhibition, plant growth promotion, plant resistance induction, and soil nutrient improvement, demonstrating potential for further applications in agriculture and forestry.

## Data availability statement

The datasets presented in this study can be found in online repositories. The names of the repository/repositories and accession number(s) can be found in the article/supplementary material.

## Author contributions

KL: Investigation, Methodology, Writing – original draft. Y-ZZ: Writing – review & editing, Formal analysis, Writing – original draft. H-YD: Formal analysis, Writing – original draft, Investigation, Methodology, Validation. Z-YW: Funding acquisition, Resources, Supervision, Writing – review & editing. P-WG: Funding acquisition, Supervision, Writing – review & editing. Z-HL: Funding acquisition, Resources, Supervision, Writing – review & editing. Z-YY: Funding acquisition, Methodology, Project administration, Writing – review & editing.

## Footnotes

^1^ http://mmit.china-cctc.org/action1.php

^2^ https://www.ncbi.nlm.nih.gov/genbank/

^3^ https://www.taobao.com/
